# Maintaining pH-dependent conformational flexibility of M1 is critical for efficient influenza A virus replication

**DOI:** 10.1038/emi.2017.96

**Published:** 2017-12-06

**Authors:** Meng-Jung Chiang, Faik N Musayev, Martina Kosikova, Zhengshi Lin, Yamei Gao, Philip D Mosier, Bashayer Althufairi, Zhiping Ye, Qibing Zhou, Umesh R Desai, Hang Xie, Martin K Safo

**Affiliations:** 1 Division of Viral Products, Office of Vaccines Research and Review, Center for Biologics Evaluation and Research, United States Food and Drug Administration, Silver Spring, MD 20993, USA; 2 Department of Medicinal Chemistry and Institute for Structural Biology, Drug Discovery and Development, School of Pharmacy, Virginia Commonwealth University, Richmond, VA 23298, USA; 3 Department of Nanomedicine and Biopharmaceuticals, National Engineering Research Center for Nanomedicine, Huazhong University of Science and Technology, Wuhan, Hubei 430074, China

**Keywords:** conformational change, influenza A virus, matrix protein 1, pH dependent, protein structure, virus replication

## Abstract

The M gene segment of influenza A virus has been shown to be a contributing factor to the high growth phenotype. However, it remains largely unknown why matrix protein 1 (M1), the major structural protein encoded by M gene, exhibits pH-dependent conformational changes during virus replication. Understanding the mechanisms underlying efficient virus replication can help to develop strategies not only to combat influenza infections but also to improve vaccine supplies. M(NLS-88R) and M(NLS-88E) are two M1 mutants differing by only a single amino acid: G88R vs G88E. G88R but not G88E was the compensatory mutation naturally selected by the virus after its nuclear localization signal was disrupted. Our study shows that, compared with M(NLS-88E) M1, M(NLS-88R) M1 dissociated quickly from viral ribonucleoproteins (vRNPs) at higher pH and took less time to dissemble *in vitro*, despite forming thicker matrix layer and having stronger association with vRNP in assembled virions. Correspondingly, M(NLS-88R) replicated more efficiently and was genetically more stable than M(NLS-88E). Crystallographic analysis indicated that M(NLS-88R) M1, like wild-type M1, is able to switch from a face-to-back-oriented conformation to a face-to-face-oriented conformation when pH drops from neutral to acidic, whereas G88E mutation causes M(NLS-88E) M1 to be trapped in a face-to-face-arranged conformation regardless of environmental pH. Our results suggest that maintaining M1 pH-dependent conformational flexibility is critical for efficient virus replication, and position 88 is a key residue controlling M1 pH-dependent conformational changes. Our findings provide insights into developing M1-based antiviral agents.

## INTRODUCTION

The viral core of influenza A virus (IAV) contains eight gene segments that are individually incorporated along with nucleoprotein (NP) into viral ribonucleoproteins (vRNPs).^1^ As the most abundant viral protein encoded by the M gene segment, matrix protein 1 (M1) forms a shell underneath a host cell-derived lipid biolayer to connect the vRNPs with the viral envelope in mature virions.^1, 2^ It has been reported that reassortment with the M gene segment of a high growth IAV, for example, A/PR/8/34 or A/WSN/33 (WSN), often generates a new virus with high growth potential,^3, 4, 5, 6^ suggesting M gene plays an important role in virus replication.

The full-length M1 protein includes 252 amino acids consisting of a highly organized N-terminal domain and a flexible but less ordered C-terminal domain that are linked by a protease-sensitive loop.^7, 8, 9^ So far, only the N-terminal domain of M1 has been structurally resolved at an atomic level, showing that at neutral pH the physiological monomers have a high tendency to oligomerize via a so-called ‘face-to-back’ arrangement,^10, 11, 12^ whereas at acidic pH the N-terminal domain exists as a dimeric structure in a so-called ‘face-to-face’ arrangement.^10, 13, 14^ Acidic conditions (pH ~5.0 or lower) have also been used to selectively isolate M1 from assembled virions.^15, 16^ These results indicate that M1 exhibits various conformations depending upon environmental pH. As the major structural protein in mature virions, the mechanism(s) by which M1 exhibits pH-dependent conformational changes during virus replication remains largely undefined.

After endocytosis, virions are internalized to late endosomes where they are exposed to low pH (≤~5.5) to induce hemagglutinin (HA)-mediated fusion with the endosomal membrane.^17, 18^ The low-pH environment inside endosomes also activates M2 ion channels embedded in the viral envelope, resulting in proton influx into the interior of the virion.^1, 19^ This leads to stepwise acidification inside the viral compartment.^20, 21^ The initial pH drop from neutral to pH ~6.0 in early endosome is believed to soften the M1 layer and make the viral envelope more pliable for membrane fusion, yet is insufficient to expose the viral core.^20, 21, 22^ The second drop to pH ~5.0–5.5 in the late endosome triggers the fusion of the viral envelope with the endosomal membrane by inducing conformational changes in HA.^20, 21, 23^ This further pH drop also causes M1 dissociation from vRNP and releases vRNP into the cytosol where they are imported into the nucleus for transcription and replication.^2, 20, 21, 24, 25^ Inhibition of M1–vRNP dissociation via acidification blockage by M2 inhibitors leads to abortive replication.^26, 27, 28^ M1 can also enter the nucleus through its nuclear localization signal motif (NLS, ^101^RKLKR^105^).^29^ By reassociation with M1, newly synthesized vRNPs are exported out of the nucleus for virus assembly and budding at the host plasma membrane.^1, 2, 24, 25^

Apparently pH-induced M1 conformational changes are dynamic and complex, and depend upon the stage of virus replication. Thus, it is difficult to fully capture these changes using biophysical techniques alone, including crystallography and transmission electron microscopy (TEM). In particular, it is unclear what controls M1 conformational changes at the atomic level following pH drops and how pH-dependent M1 conformational changes affect virus replication. Elucidating the mechanisms underlying efficient virus replication is the prerequisite for the development of effective countermeasures against both seasonal and pandemic influenza.

Both M(NLS-88R) and M(NLS-88E) are M1 mutants in the WSN background that differ in a single mutation at M1 position 88 (G88R vs G88E).^30^ G88R but not G88E was originally acquired by the virus as a spontaneous compensatory mutation after NLS disruption.^30^ As a result, M(NLS-88R) not only has a thick M1 layer in assembled virions but also replicates efficiently *in vitro* and *in vivo*, similar to the parent virus WSN.^30, 31^ In contrast, M(NLS-88E) has a thin M1 layer in mature virions and replicates inefficiently *in vitro* and *in vivo*.^30^ Using M(NLS-88R) and M(NLS-88E) as model viruses, we revealed that the transition from a face-to-back orientation at neutral pH to a face-to-face orientation at acidic pH allows M1 to quickly dissociate from vRNP for efficient replication. However, the G88E mutation causes M1 to be trapped in a face-to-face orientation regardless of pH that consequently resulted in inefficient virus growth and genetic instability. Our results suggest that maintaining M1 pH-dependent conformational flexibility is critical for efficient IAV replication, and position 88 serves as a ‘switch’ that controls M1 pH-dependent conformational changes. Our findings provide structural insights for the development of M1-based antiviral agents.

## MATERIALS AND METHODS

### Viruses

The wild-type (wt) WSN and M1 triple mutants M(NLS-88R) and M(NLS-88E), bearing R101S and R105S in NLS ^101^RKLKR^105^ and an additional mutation G88R or G88E in the adjacent region, were generated by reverse genetics.^30^ Except the three mutations in the M1 gene, the other seven gene segments in M1 triple mutants remain the same as WSN. All the viruses were continuously propagated in 9–10-day-old embryonated eggs at 33 °C for up to 12 passages. Individual gene segments of each virus passage were full-length sequenced (Center for Biologics Evaluation & Research/Food and Drug Administration (CBER/FDA) core facility). Unless otherwise specified, only viruses of egg passages 2 or 3 were used for *in vitro* replication, confocal microscopy and TEM experiments.

### *In vitro* replication

Madin-Darby canine kidney (MDCK) cells (CBER/FDA) in 80–90% confluence were infected with WSN or M1 triple mutants of egg passage 2 at a multiplicity of infection of 0.002 at 33 °C, 5% CO_2_ for up to 72 h.^30, 31, 32^ Infectious viral particles sampled at different time points were titrated by a plaque assay.

### Cellular membrane binding assay

Cellular membrane-associated M1 was assessed as described previously with minor modifications.^30, 33^ Briefly, MDCK cells after 24 h post infection (multiplicity of infection=2) were dissociated in the hypotonic buffer containing 1 mM Tris-HCl (pH 7.4) and 0.1 mM MgCl_2_ on ice for 60 min. After being passed through a 20-gauge needle at least 20 times, cell homogenates were centrifuged at 1000 × *g*, 4 °C for 10 min. The post-nuclear supernatants were subjected to ultracentrifugation at 100 000 × *g*, 4 °C for another 60 min to collect the pellets containing the cellular membranes. The aliquots of the cellular membranes were separately incubated in pH 7.4 or pH 5.5 phosphate-buffered saline (PBS) buffer at 37 °C for 30 min. The pH-treated cellular membranes were then subjected to ultracentrifugation again. The pellets were collected and analyzed under reducing condition by western blot using β-actin as the control. Biotin-conjugated anti-M1 antibody (Abcam ab20351, Cambridge, MA, USA) and mouse anti-β-actin antibody (Santa Cruz sc-47778, Dallas, TX, USA) were used for probing followed by IRDye-680LT-labeled streptavidin (LI-COR 926–68031, Lincoln, NE, USA) and IRDye-800CW-labeled donkey anti-mouse (LI-COR 926–32212). The blots were imaged and analyzed using an Odyssey imaging system (LI-COR, Lincoln, NE, USA).

### Acid-mediated bypass and confocal microscopy

WSN, M(NLS-88R) or M(NLS-88E) of egg passage 3 was concentrated by ultracentrifugation (30 000 rpm × 90 min, 4 °C). Aliquots of concentrated viruses were preincubated in pH-adjusted PBS buffer at 37 °C for 60 min and were then neutralized by an equal volume of Opti-MEM I medium (Thermo Fisher, Gaithersburg, MD, USA) before cell infection. Prechilled MDCK cells seeded on Millicell EZ SLIDE 8-well glass slides (Millipore, Temecula, CA, USA) were washed once with cold Opti-MEM, and pH-treated viruses (multiplicity of infection of 100) were added followed by 30 min of incubation on ice to allow virus binding. After removing unbound viruses, cells were pulsed in warm Dulbecco’s modified Eagle’s medium (Thermo Fisher) containing 50 mM citrate buffer (pH 5.0, fusion medium) at 37 °C for 2–3 min to bypass the endocytic pathway and allow direct membrane fusion.^21^ After two brief washes with cold Opti-MEM, cells were immediately incubated in warm Dulbecco’s modified Eagle’s medium containing 50 mM HEPES and 20 mM NH_4_Cl (pH 7.4, stop medium) at 37 °C to block endosome acidification.^21^ After 30 min, cells were washed and fixed with 4% paraformaldehyde (Sigma, St Louis, MO, USA) followed by permeabilization in PBS buffer containing 0.5% Triton X-100 and 0.2% bovine serum albumin (Sigma). Infected cells were then labeled with biotin-conjugated anti-M1 polyclonal antibody (Abcam ab20351) and/or NP-specific mouse monoclonal antibodies (Millipore MAB8257 and MAB8258 mixed in 1:1, v/v) diluted 1:1000 in SEA BLOCK blocking buffer (Thermo Fisher). Secondary antibodies included Alexa Fluor 488 FluoroNanogold Streptavidin (Invitrogen A-24926, Carlsbad, CA, USA) and/or Alexa Fluor 594-conjugated goat anti-mouse IgG (H+L) (Invitrogen A-11020). Immunofluorescent stained slides were then mounted using Fluoromount-G with 4',6-diamidino-2-phenylindole (eBioscience, San Diego, CA, USA), and the images were acquired using FluoView FV10i Confocal Laser Scanning Microscope (Olympus, Waltham, MA, USA). Captured images were reprocessed for enhanced view on NP and M1 staining spots using Imaris Image Analysis Software (Bitplane, Concord, MA, USA). Colocalized M1–NP spots were counted by three individuals and were expressed as the percentages of total (M1+NP) spots. Statistical difference (*P*<0.05) vs the same virus pretreated with pH 7.4 was determined using one-way analysis of variance (GraphPad Prism 6, La Jolla, CA, USA).

### Transmission electron microscopy

WSN, M(NLS-88R) or M(NLS-88E) of egg passage 3 was concentrated by ultracentrifugation (30 000 rpm × 90 min, 4 °C) followed by a discontinuous 15–30–60% sucrose gradient purification (27 000 rpm × 90 min, 4 °C).^30^ Aliquots of purified viruses were incubated in pH 7.4 or pH 5.5 PBS buffer at 37 °C for 10 min, respectively. Following pH treatment, viruses were fixed with 2% paraformaldehyde and 2% glutaraldehyde (Sigma) in PBS at room temperature overnight followed by post-fixation with 1% osmium tetroxide for another 1 h. After dehydration and infiltration, fixed viruses were embedded in epoxy resin. Ultrathin sections were stained with uranyl acetate and lead citrate and were examined under a Zeiss EM 912 transmission electron microscope (TEM) (Thornwood, NY, USA) equipped with a Keenview digital camera (Olympus). Approximately 100 virions per virus per pH treatment were blindly counted by two individuals and were expressed as a percentage of partially spiked virions per treatment based on the morphology observed under TEM. The M1 layer thickness of individual virions was determined by averaging the measures at the clockwise positions 3, 6, 9 and 12 o’clock under TEM, respectively.^30^ Ten representative virions per virus per pH treatment were measured.

### Recombinant M1 protein

The His-tagged N^1-165^-domain of M1 in pET30a vector (Novagen, Temecula, CA, USA) was generated and overexpressed as described previously.^10^ All recombinant M1 proteins were purified by affinity chromatography in combination with fast protein liquid chromatography columns with the purity >90% by SDS–polyacrylamide gel electrophoresis analysis (Cellomics, Halethorpe, MD, USA). Purified recombinant M1 proteins were kept in 55 mM KH_2_PO_4_/K_2_HPO_4_/H_3_PO_4_, 0.2 M NaCl and 2 mM TCEP (tris(2-carboxyethyl)phosphine) buffer at pH 4.0 and stored at −20 °C until use.

### M1–M1 interactions under different pH treatments

Biolayer interferometry (BLI) is a label-free technique that detects macromolecular interactions by assessing the interference patterns of white light reflected from the surface of a biosensor tip. A change in the thickness of molecules bound to immobilized ligand on the biosensor tip causes a wavelength shift: more molecules bound, bigger shifts in the wavelength. The M1–M1 interactions under different pH treatments were determined using an Octet Qke biolayer interferometer equipped with streptavidin biosensor tips (ForteBio, Inc., Menlo Park, CA, USA). Recombinant M1 proteins were freshly dialyzed against PBS pH 7.4 (1:1000, v/v) three times. Aliquots of dialyzed M1 proteins were biotinylated with NHS-PEG4-Biotin (Thermo Scientific, Frederick, MD, USA) in a 1:1 molar ratio at room temperature for 30 min followed by removal of excessive biotin with 7K Zeba desalt spin column (Thermo Scientific). Meanwhile, aliquots of dialyzed M1 proteins without biotinylation were serially diluted in PBS solution containing 0.01% bovine serum albumin and 0.002% Tween-20 (kinetics buffer) with pH adjusted to 7.4 or 5.5 by 2 M citric acid. Biotinylated M1 was loaded onto streptavidin biosensors at 0.2 μg/ml in pH 7.4 kinetics buffer to a maximum response of ~1 nm. Binding of unbiotinylated M1 in pH 7.4 or pH 5.5 kinetics buffer was measured for 200 s. The measure cycle was maintained at 30 °C and 1000 rpm. The first 120 s association data were processed and analyzed by nonlinear regression curve fitting (association kinetics—two or more concentrations of hot) using GraphPad Prism 6.

### Crystallization of M1 triple mutant proteins

The truncated N^1–165^-domain of M(NLS-88R) M1 protein was dialyzed against 55 mM K_2_HPO_4_/KH_2_PO_4_/H_3_PO_4_, 0.2 M NaCl, 2 mM TCEP (pH 4.0) and concentrated to 13.7 mg/ml. Crystallization of M(NLS-88R) M1 protein was carried out by the hanging-drop vapor diffusion method with commercially available screening kits at 20 °C. X-ray quality crystals were obtained from a reservoir solution containing 75 mM Tris-HCl (pH 7.0) and 15–17.5% PEG-1000. As the crystallization drop was estimated to be pH 5.5, the crystal and the ensuing crystal structure is referred to as M(NLS-88R)-acidic. A second crystal form of M(NLS-88R) M1 protein was obtained with 10 mg/ml of protein dialyzed in 50 mM K_2_HPO_4_/KH_2_PO_4_, 0.2 M NaCl, 10 mM bME (pH 7.2) and a reservoir solution containing 0.2 M NaF, 25% PEG-3350 (pH 7.3) at 20 °C. This crystal and the subsequent structure are referred as M(NLS-88R)-neutral as the final crystallization drop was at pH 7.3.

The truncated N^1-165^-domain of M(NLS-88E) M1 protein was dialyzed in 25 mM Na/Hepes buffer (pH 7.0), 0.2 M NaCl, and 2 mM TCEP and concentrated to 16.7 mg/ml, and stored in Eppendorf tubes at 4 °C that interestingly crystallized 2 weeks later. This crystal and the subsequent crystal structure is referred to as M(NLS-88E)-neutral as the final crystallization condition was at pH 7.0. A second crystal form of M(NLS-88E) was obtained by the sitting-drop vapor diffusion method with 8 mg/ml of protein (dialyzed in 55 mM K_2_HPO_4_/KH_2_PO_4_/H_3_PO_4_, 0.2 M NaCl, 2 mM TCEP, pH 3.2) and a reservoir solution containing 8% PEG-8000, 100 mM Tris-HCl (pH 8.2). The crystallization drop was estimated to have a pH of 6.2, and therefore the ensuing crystal structure is referred to as M(NLS-88E)-acidic.

### Data collection and structure determination

For X-ray data collection, crystals of M(NLS-88R)-acidic were cryoprotected in reservoir buffer supplemented with 30% PEG-1000, whereas M(NLS-88R)-neutral crystals were cryoprotected in 0.2 M NaF and 30% PEG-3350 solution with subsequent annealing in 25 mM K_2_HPO_4_, 40% PEG-3350, 186 mM NaCl and 61 mM NaF, pH 7.3. M(NLS-88E)-neutral crystals were cryoprotected in 25 mM Na-Hepes (pH 7.0), 3 M NaCl, 5% glycerol and 2 mM TCEP solution, whereas M(NLS-88E)-acidic crystals were cryoprotected by first washing in a mother liquid solution and then transferring stepwise to similar solutions containing 10, 20 and 25% glycerol. X-ray data sets of all mutant crystals were obtained at 100 K on an R-axis IV++ image plate detector using CuKα X-ray (λ=1.5418) from a Rigaku Micro-Max-007 X-ray source equipped with Varimax confocal optics operating at 40 kV and 20 mA (Rigaku, The Woodlands, TX, USA). Crystals of M(NLS-88R)-acidic, M(NLS-88R)-neutral, M(NLS-88E)-acidic and M(NLS-88E)-neutral diffracted to 2.0 Å, 3.0 Å, 2.5 Å and 2.5 Å resolutions, respectively, and the data sets were processed and scaled with Rigaku D*TREK software. Diffraction data statistics are shown in Table 1.

All four M1 mutant structures were determined using molecular replacement with the program PHENIX v.1.9_1692.^34^ M(NLS-88R)-acidic and M(NLS-88R)-neutral structures were solved using the wt-M1 dimer structure 1AA7 (crystallized at acidic pH)^13^ and wt-M1 monomer structure 1EA3 (crystallized at neutral pH),^12^ respectively. The two M(NLS-88E) structures were also solved using 1EA3. Refinement and model building were carried out using PHENIX^34^ and COOT.^35^ Final refinement of M(NLS-88R)-acidic, M(NLS-88R)-neutral, M(NLS-88E)-acidic and M(NLS-88E)-neutral resulted in *R*_work_/*R*_free_ of 19.2/23.6, 27.2/32.1, 22.0/31.2 and 22.8/30.8, respectively. Refinement statistics are summarized in Table 1. The quaternary differences were quantified by least-squares superposition of the dimeric structures and/or by superposition of the respective monomers A followed by determination of the screw rotation that superposed the respective monomers B. The relative stabilities of the different dimeric arrangements were determined by calculating the buried surface areas using proximal isovelocity surface area (PISA) method.

## RESULTS

### Crystal structure determination

We and others have reported that wt-M1 can access multiple conformational and/or oligomeric states depending on the environmental pH.^10, 12, 13, 36^ We then determined whether M(NLS-88R) and M(NLS-88E) M1 also showed pH-dependent conformations by X-ray crystallography. Detailed crystallographic data for all four structures (M(NLS-88R)-neutral, M(NLS-88R)-acidic, M(NLS-88E)-neutral and M(NLS-88E)-acidic) obtained are presented in Table 1.

**Figure 1:**
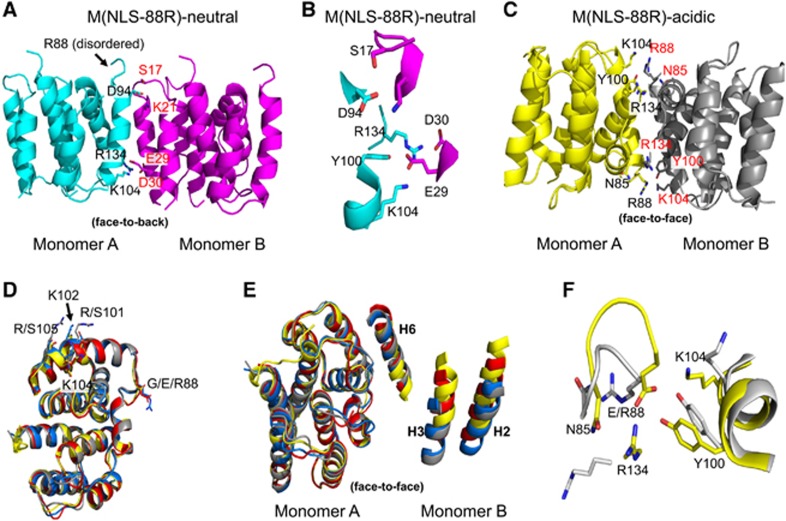
Monomeric and dimeric arrangements of matrix protein 1 (M1) structures. (**A**) Monomer–monomer oligomerization in M(NLS-88R)-neutral. (**B**) Detailed monomer–monomer contacts in M(NLS-88R)-neutral. (**C**) Dimer structure of M(NLS-88R)-acidic. (**D**) Comparison of monomer structures of wild-type (wt) 1EA3 (gray, neutral), M(NLS-88R)-acidic (blue), M(NLS-88E)-acidic (red) and M(NLS-88E)-neutral (yellow). (**E**) Comparison of dimer structures of wt 1AA7 (gray, acidic), M(NLS-88R)-acidic (blue), M(NLS-88E)-acidic (red) and M(NLS-88E)-neutral (yellow). (**F**) Comparison of the two symmetry-related dimer interfaces of M(NLS-88R)-acidic (gray) and M(NLS-88E)-neutral (yellow).

M(NLS-88R)-neutral occurs as physiological monomers similar to wt-M1 structure 1EA3 (also crystallized at neutral pH),^12^ and are arranged loosely in a so-called ‘face-to-back’ orientation with each other in the cell (Figure 1A). The residues Lys104 (part of the NLS motif), Arg134, Tyr100 and Asp94 on the ‘face’ of one molecule (Monomer A) interact with several complementary residues on the ‘back’ of a second molecule (Monomer B), including Glu29, Asp30, Lys21 and Ser17 (Figures 1A and 1B). Despite also crystallizing at a neutral pH, the two monomers (A and B) of M(NLS-88E)-neutral unexpectedly associate to form a physiological face-to-face dimer with the NLS basic residues adjacent to each other similar to wt-M1 dimer 1AA7 (crystallized at acidic pH).^13^ The two monomers of M(NLS-88E)-neutral are similar with a root mean square deviation (r.m.s.d.) of ~0.4 Å, but are different from those in wt-M1 1AA7.^13^

Both M(NLS-88R)-acidic and M(NLS-88E)-acidic occur as physiological dimers as expected for low-pH structures.^10, 13, 14^ M(NLS-88R)-acidic has monomers A and B or C and D associated to form two independent physiological dimers (dimers 1 and 2, respectively) in the crystal. Least-squares superpositions of the four M(NLS-88R)-acidic monomers or superposition of its two independent dimers resulted in low r.m.s.d.s of 0.2–0.4 Å or 0.4 Å, suggesting similar quaternary conformation or monomer–monomer arrangements. Similar to wt-M1 dimer 1AA7 crystallized at acidic pH,^13^ the monomers in each dimer of M(NLS-88R)-acidic are arranged in a so-called ‘face-to-face’ orientation that are related by a non-crystallographic twofold axis with the basic residues of the NLS motif close to the dimer interface (Figure 1C). On the other side, M(NLS-88E)-acidic has monomers A and B associate in a face-to-face arrangement to form dimer 1, and has molecule C similarly associated with its dyad equivalent to form dimer 2. All three M(NLS-88E)-acidic monomers superposed on each other with a r.m.s.d. of ~0.5 Å. However, least-squares superposition of the two M(NLS-88E)-acidic independent dimers 1 and 2 resulted in a very large r.m.s.d. of 1.2 Å (see Supplementary Table S2 in the Supplementary Material), indicating that the two dimers of M(NLS-88E)-acidic show significantly different dimer arrangements.

**Figure 2:**
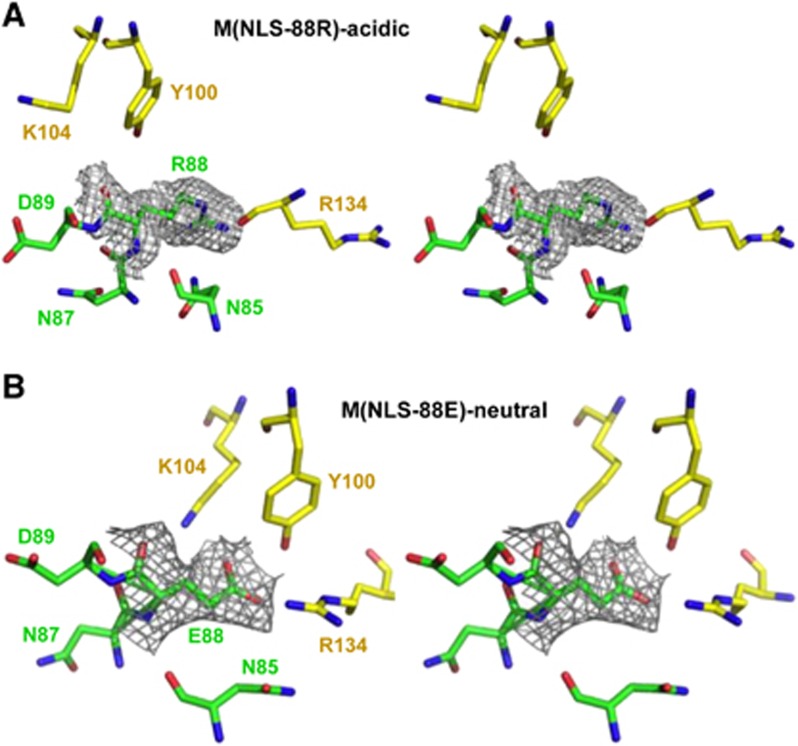
Stereo-view of electron density map and final dimer model of residue 88 environment in M(NLS-88R)-acidic and M(NLS-88E)-neutral. The maps are contoured at 1.0α. Monomers A and B that form the face-to-face dimer are colored yellow and green, respectively. (**A**) Dimer interface of M(NLS-88R)-acidic. (**B**) Dimer interface of M(NLS-88E)-neutral.

In general, the monomeric fold in each structure is similar and consists of two domains as previously reported:^11^ an N-terminal domain of helices H1, H2, H3 and H4 connects with a C-terminal domain of helices H6, H7, H8 and H9 by a helix-containing H5 linker (Figure 1D). Interactions between the monomers that form the face-to-face dimer in M(NLS-88R)-acidic, M(NLS-88E)-acidic or M(NLS-88E)-neutral involve hydrophobic contacts (mainly between the symmetry-related helices H6, and between helices H9 and loops L9) (Figure 1E), as well as several hydrogen-bond and/or salt-bridge interactions (see Supplementary Table S3 in the Supplementary Material). A few dimer interface hydrogen-bond interactions (Asn85 to Arg134 and X88 to Tyr100) are conserved, whereas the rest of the contacts are mostly unique to each structure (Figure 1F; see Supplementary Table S3), highlighting the differences in the relative monomer–monomer arrangement of different dimers. The analyses indicated that M(NLS-88R)-acidic (dimers 1 and 2) had a very similar dimeric arrangement as wt-M1 1AA7 structure (r.m.s.d. of 0.6 Å screw rotation angle of 2°, Figure 2A and Supplementary Table S2). However, M(NLS-88E)-neutral dimer differs significantly from wt-M1 1AA7 or M(NLS-88R)-acidic (r.m.s.d. of 1.9 Å screw rotation angle of ~17°, Figure 2B and Supplementary Table S2). Dimer 1 of M(NLS-88E)-acidic is more like M(NLS-88E)-neutral dimer (r.m.s.d. of ~0.6 Å screw rotation angle of ~8°, see Supplementary Table S2), but not its dimer 2 (r.m.s.d. of ~0.6 Å screw rotation angle of ~15°, see Supplementary Table S2). In addition, the buried surface area of M(NLS-88R)-acidic (2327 Å^2^) or wt-M1 1AA7 (2187 Å^2^) is almost twice of those in M(NLS-88E)-neutral (1264 Å^2^) and M(NLS-88E)-acidic (1279 Å^2^ for dimer 1 and 1382 Å^2^ for dimer 2) (see Supplementary Table S3), indicative of different dimer stabilities.

### pH-dependent M1–M1 interactions

**Figure 3:**
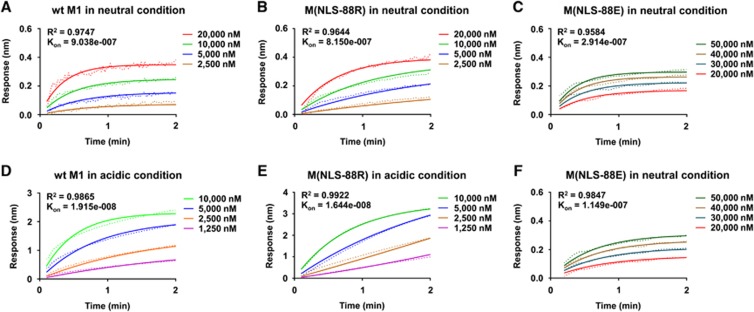
The matrix protein 1 (M1)–M1 interactions under different pH treatments assessed by biolayer interferometry (BLI). The recombinant N^1-165^-domain of wild type (wt), M(NLS-88R) or M(NLS-88E) M1 protein was used to assess M1–M1 interactions in neutral (pH 7.4) or acidic condition (pH 5.5) by BLI. (**A** and **D**) The wt-M1–M1 interactions in neutral and acidic conditions; (**B** and **E**) M(NLS-88R) M1–M1 interactions in neutral and acidic conditions; (**C** and **F**) M(NLS-88E) M1–M1 interactions in neutral and acidic conditions. The association rate (*K*_on_) and *R*^2^ value after nonlinear regression curve fitting (association kinetics–two or more concentrations of hot) are shown (*n*=2–7 replicates/concentration).

We then determined M1–M1 interactions under different pH conditions by BLI. Regardless of pH conditions, M(NLS-88R) had M1–M1 interactions in a dose-dependent manner similar to wt-M1 (Figure 3A vs 3B and Figure 3D vs 3E). Despite in lower protein concentrations (1250–10 000 nM), the BLI association signals of M(NLS-88R) M1 in acidic condition were tighter than the signals at neutral pH with higher protein concentrations (2500–20 000 nM) (Figure 3B *K*_on_=8.150e−007 vs Figure 3E *K*_on_=1.644e−008). This indicated that the M1–M1 interactions in the face-to-face-arranged M(NLS-88R) dimeric conformation (acidic condition) were stronger than those in the face-to-back-arranged M1(NLS-88R) monomer–monomer conformation (neutral condition). Unlike M(NLS-88R), M(NLS-88E) M1 protein showed detectable signals at either pH only when the protein concentrations increased to 20 000–50 000 nM (Figures 3C and 3E). Under both pH conditions, the BLI association signals of M(NLS-88E) M1 were comparable, but still 10-fold weaker than that of M(NLS-88R) in acidic condition, though close to that of M(NLS-88R) at neutral pH (Figure 3C vs 3B and Figure 3F vs 3E). These results suggested that M(NLS-88E) M1 (face-to-face-arranged dimers in both neutral and acidic conditions) had much weaker M1–M1 interactions than M(NLS-88R) M1 in neutral condition (face-to-back-oriented monomer–monomer) or in acidic condition (face-to-face-arranged dimer).

### Cellular membrane-associated M1 under different pHs

During virus assembly and budding, M1 is associated with host cellular membrane through its N terminus.^2, 37, 38, 39^ Thus, we investigated the effects of different pHs on cellular membrane-associated M1. The results were consistent with our previous report that M(NLS-88E) had much less M1 associated with host cellular membrane than M(NLS-88R) or WSN (Supplementary Figure S1). However, cellular membrane-associated M1, regardless of viruses, was apparently not affected by different pH treatments (Supplementary Figure S1). This result indicated that M1 pH-dependent conformational changes unlikely occurred during virus assembly.

### Acidic pH-induced morphology changes in purified virions

**Figure 4:**
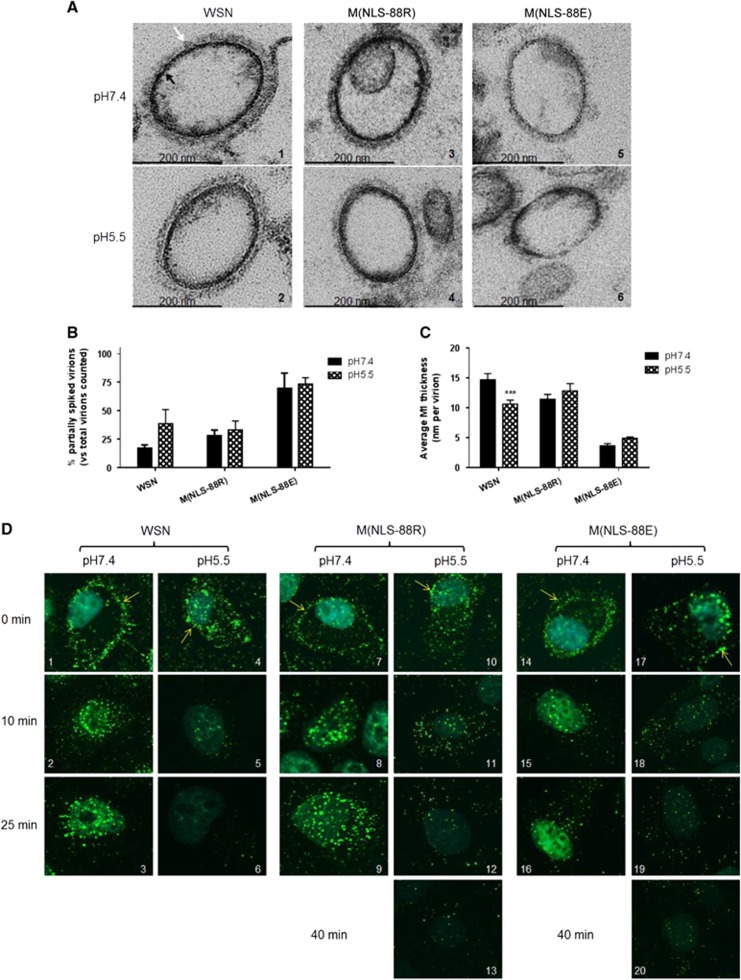
Virion morphology changes and matrix protein 1 (M1) cytoplasmic disintegration under different pH treatments. (**A**) Representative transmission electron microscopy (TEM) images of purified wild-type A/WSN/33 (WSN), M(NLS-88R) or M(NLS-88E) pretreated with the indicated pH buffers. Images were acquired under a Zeiss EM 912 transmission electron microscope equipped with a Keenview digital camera. Black and tan arrows indicate M1 layer and spikes, respectively. (**B**) The percentages of partially spiked virions per ∼100 viral particles blindly counted by two individuals. The data are expressed as the average±SEM. (**C**) The average M1 thickness in different pH pretreated virions under TEM (*n*=10 virions). ***P<0.001 by two-way analysis of variance (ANOVA) vs the same virus pretreated with pH 7.4. (**D**) The M1 cytoplasmic disintegration of different pH pretreated WSN, M(NLS-88R) or M(NLS-88E). Immunofluorescent stained M1 (green) was visualized under FluoView FV10i Confocal Laser Scanning Microscope.

The low pH (≤~5.5) environment inside endosomes not only triggers HA-mediated membrane fusion,^17, 18^ but also induces solubilization of viral envelope.^15, 16, 24, 40^ As M(NLS-88R) and M(NLS-88E) have different M1 layer thicknesses in mature virions,^30^ we then investigated whether this difference affected viral envelope solubilization after brief acidic exposure. In general, the TEM images indicated that the mature M(NLS-88R) virion had very similar morphology to WSN virions regardless of pH treatment (Figures 4A1 and 4A4): the majority of viral particles were fully covered by well-organized HA spikes (indicated by white arrows), and the M1 layers (indicated by black arrows) appeared as dark thick lines evenly distributed along the viral membrane. In contrast, ∼35% of M(NLS-88E) virions were covered by a thin coat of HA spikes and the rest were partially spiked at both neutral and acidic conditions (Figures 4A5 and 4A6 and Figure 4B). The M1 layer of M(NLS-88E) virions also appeared disrupted and was much thinner than that of M(NLS-88R) viral particles at both pH conditions (Figures 4A5 and 4A6 and Figure 4C). A brief acidic treatment (pH 5.5 × 10 min) had no obvious effects on the morphology of M(NLS-88R) or M(NLS-88E) viral particles, though it considerably increased the percentage of partially spiked WSN virions and significantly reduced their M1 thickness (Figures 4B and 4C). Virions exposed to pH 5.0 or lower were excluded from the morphology experiment, because they became so fragile that the majority had surface glycoproteins depleted during the TEM multiple fixations/dehydration process, making it difficult to determine whether acidic treatment was the main cause of morphological changes observed. Nevertheless, the TEM results confirmed that a brief acidic exposure indeed solubilizes the viral envelope of WSN. However, they also suggested that the M1 layer of M(NLS-88R) or M(NLS-88E), regardless of thickness, was less sensitive to mild acidic conditions as that of WSN.

### pH-dependent M1 disintegration

We next investigated whether the M1 layer thickness also affected the M1 disassembly inside infected cells. Acid bypass is an approach that temporarily decreases extracellular pH to induce HA fusion at the plasma membrane without involving endosomes.^20, 21^ Following the induction of acid bypass, the M1 (green) from pH 7.4 pretreated WSN, M(NLS-88R) or M(NLS-88E) mainly accumulated along the cytoplasmic membrane (indicated by yellow arrows) and then slowly translocated into the cytoplasm after 25 min of incubation (Figures 4D1, 4D2, 4D3, 4D7, 4D8, 4D9, 4D14, 4D15 and 4D16). In contrast, pH 5.5 pretreated WSN or M(NLS-88R) had substantial M1 already translocated into the cytoplasm immediately upon the induction of acid bypass (Figures 4D4 and 4D10). Unlike pH 5.5 pretreated WSN that had the cytoplasmic M1 quickly disappear within 25 min post acid bypass, the cytoplasmic M1 signal of pH 5.5 pretreated M(NLS-88R) declined more slowly and took ∼40 min to fade out (Figures 4D5, 4D6, 4D11, 4D12 and 4D13). However, the cytoplasmic M1 signal of pH 5.5 pretreated M(NLS-88E) retained even longer than that of pH 5.5 pretreated M(NLS-88R) and showed no obvious fading at 40 min post acid bypass (Figure 4D18, 4D19 and 4D20). These results showed that the thin M1 layer of M(NLS-88E) actually disassembled more slowly in cytoplasm than the thick M1 layer of M(NLS-88R) or WSN after acidic exposure.

### pH-dependent M1–NP colocalization

**Figure 5:**
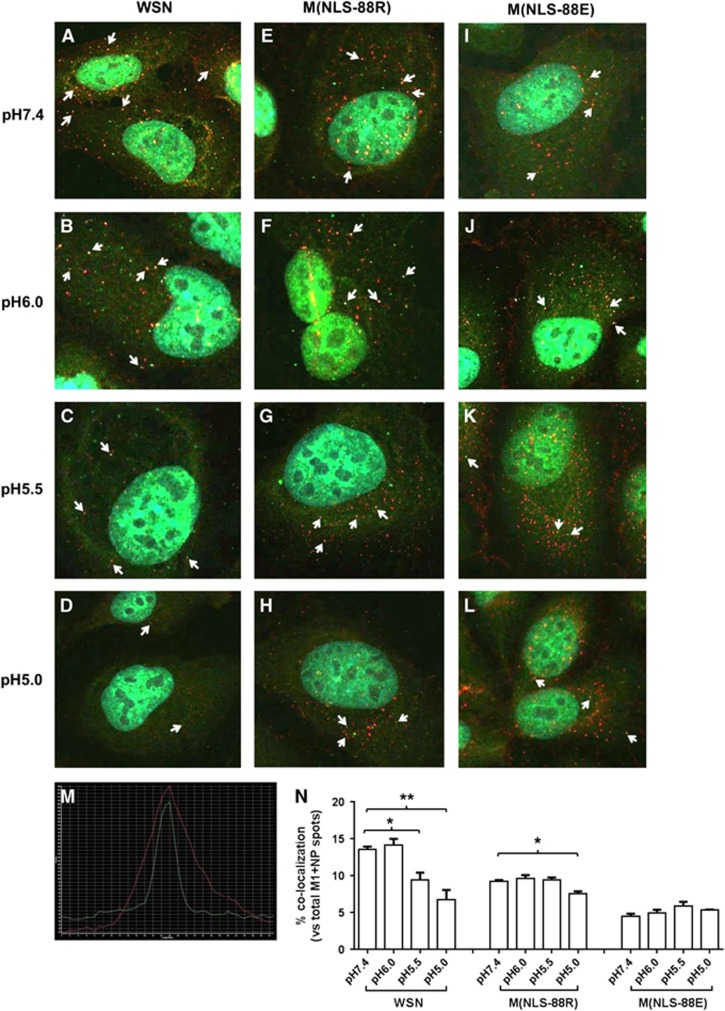
pH-dependent matrix protein 1–nucleoprotein (M1–NP) co–localization. Viruses pretreated with different pH conditions were bound to prechilled Madin-Darby canine kidney (MDCK) cells on ice for 60 min followed by acid bypass to allow direct membrane fusion (Methods). (**A**–**L**) M1–NP co-localization of pH-treated wild-type A/WSN/33 (WSN), M(NLS-88R) or M(NLS-88E) in MDCK cells. Immunofluorescent stained M1 (green) and NP (red) were visualized under FluoView FV10i Confocal Laser Scanning Microscope. (**M**) Representative histogram overlay of colocalized M1 and NP. Arrows indicate confirmed M1–NP colocalization by histogram overlay. (**N**) The percentages of colocalized M1–NP spots vs the total (M1+NP) spots in (**A**–**L**) (*n*=3 individual counts/virus/treatment). *P<0.05 and **P<0.01 by one-way analysis of variance (ANOVA), respectively.

In addition to induction of HA-mediated membrane fusion,^17, 18^ acidic exposure also causes vRNP to dissociate from M1 for subsequent nuclear import.^24^ Recent TEM studies have also suggested that M1 may undergo conformational changes before M1–vRNP dissociation.^23, 40^ As M(NLS-88R) showed stronger M1–vRNP association in assembled viral particles than M(NLS-88E),^30^ presumably it would be more difficult to break M1–vRNP association in M(NLS-88R) virions than in M(NLS-88E) virions. To confirm this, we assessed the effects of acidic pHs on M1–NP colocalization in virus-infected MDCK cells via acid bypass to allow direct release of vRNP into the cytoplasm without involving the endocytic pathway.^20, 21^ Figure 5 shows the M1–NP colocalization in virions treated with different pHs. The same confocal images were also reprocessed for enhanced view and spot counting using Imaris Image Analysis Software (Supplementary Figure S2). When the pH dropped from 7.4 to 5.0, WSN had noticeably weakened M1–NP colocalization (13.6±0.4% at pH 7.4 vs 9.4±1.0% at pH 5.5 or 6.7±1.3% at pH 5.0, *P*<0.05, Figure 5N). M(NLS-88R) appeared less sensitive to acidic pH and showed no significantly reduced M1–NP colocalization only after the pH dropped below 5.5 (9.2±0.2% at pH 7.4 vs 7.5±0.3% at pH 5.0, *P*<0.05, Figure 5N). In contrast, M(NLS-88E) showed no changes in the percentage of colocalized M1–NP between pH 7.4 and pH 5.0, despite having the lowest overall M1–NP colocalization among three viruses (Figure 5N). These results suggested that M(NLS-88R), in spite of a stronger M1–vRNP association than that in M(NLS-88E), was more sensitive to pH drops whereby M1–vRNP dissociation occurred at a higher pH.

### *In vitro* replication and genetic stability of M1 mutants

**Figure 6:**
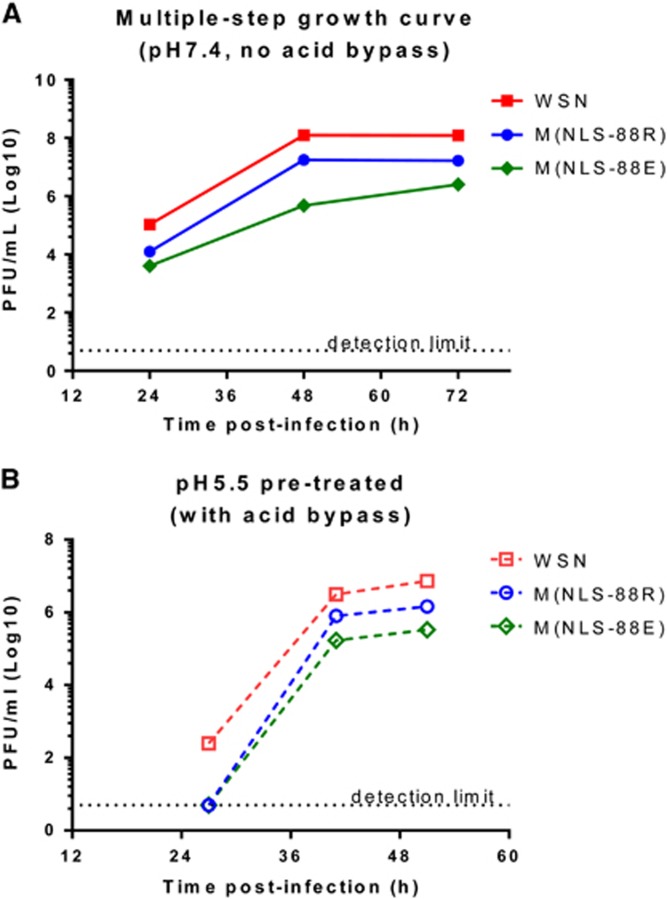
The *in vitro* replication of matrix protein 1 (M1) triple mutants after acid treatment. (**A**) *In vitro* multiple-step replication of wild-type A/WSN/33 (WSN), M(NLS-88R) and M(NLS-88E) in Madin-Darby canine kidney (MDCK) cells at pH 7.4 without acid bypass. (**B**) The replication kinetics of pH 5.5 pretreated M1 mutants in MDCK cells with acid bypass. All replication experiments had been repeated multiple times. Data (*n*=2 replicates/virus/time point) from representative experiments are shown.

The multiple-step growth curves at pH 7.4 confirmed that M(NLS-88R) replicated more efficiently than M(NLS-88E) (Figure 6A).^30^ Both M(NLS-88R) and M(NLS-88E) have identical HA gene,^30^ but have different amounts of HA spikes on viral surface (Figure 4A). However, acid bypass allows direct cytoplasmic release of vRNP without concerning the differences in HA.^20, 21^ As shown in Figure 6B, the *in vitro* growth of pH 5.5 pretreated M(NLS-88R) remained higher than that of M(NLS-88E) after induction of acid bypass. These replication results appeared to correlate with the observations of pH-dependent M1 disintegration and M1–vRNP dissociation. Furthermore, M(NLS-88R) was genetically more stable than M(NLS-88E) (see Supplementary Table S1 in the Supplementary Material). Unlike M(NLS-88E) that acquired 3 additional mutations in the M1 gene after four serial egg passages, M(NLS-88R) gained no additional mutations in the M1 gene until egg passage 11 (see Supplementary Table S1).

## DISCUSSION

Our previous study indicated that M(NLS-88R) not only possessed much thicker M1 layer but also had stronger M1–vRNP association in viral particles than M(NLS-88E) virions.^30^ Thus, it would presumably be more difficult to disrupt the M1 layer in M(NLS-88R) virions than that in M(NLS-88E) virions. However, M(NLS-88R) showed a faster cytoplasmic M1 disassembly than that of M(NLS-88E) after pH 5.5 pretreatment, suggesting M(NLS-88R) M1 is more sensitive to acidic pH than M(NLS-88E) M1, irrespective of M1 layer thickness. Furthermore, M(NLS-88E) had a more difficult M1–vRNP dissociation than M(NLS-88R) in response to pH drops, clearly indicating that this was also irrelevant to a weaker physical binding strength between M1 and vRNP. In addition, the faster cytoplasmic M1 disintegration and more facile M1–vRNP dissociation after mild acidic exposure correlated well with more efficient replication of M(NLS-88R). Moreover, our current study also showed that cellular membrane-associated M1 was not affected by environmental pHs. These results taken together suggest that acidic pH-induced M1 conformational changes are likely to play a role in M1–vRNP dissociation, rather than to affect virus assembly during viral life cycle. This is consistent with recent TEM studies that M1 may undergo conformational changes before dissociation from vRNP.^23, 40^

Although differing by a single amino acid (G88R vs G88E), M(NLS-88R) M1 and M(NLS-88E) M1 respond to environmental pH differently as indicated by the crystal structures obtained in the current study. M(NLS-88R) M1, similar to wt-M1, is able to switch from a face-to-back monomer–monomer association at neutral pH to a face-to-face-oriented dimeric conformation at acidic pH. However, M(NLS-88E) M1 is trapped in a face-to-face-oriented conformation irrespective of environmental pH. The face-to-back monomeric arrangement of M1 occurring at neutral pH is likely necessary for the formation and maintenance of a confluent and strong matrix layer underneath the viral envelope, whereas the face-to-face dimeric arrangement at acidic pH may describe M1 that has dissociated from the extended matrix layer.^10, 13, 14^ Consistent with previous reports,^10, 13, 14, 15, 16^ we have observed that the N^1–165^-domain of wt M1 purified only under acidic conditions retains dimeric conformation. The C-terminal his-tagged M1 N^1–170^-domain made by Zhang *et al.*^36^ was purified at pH 7.9 that was found to completely dissociate into monomers in acidic solution.^36^ Interestingly, Zhang *et al.*^36^ also reported that intact M1 existed as stable dimers in acidic solution.^36^ Of note, the face-to-face dimeric interaction is stronger than the face-to-back monomeric interaction as indicated by the BLI data. Thus, the acid-facilitated M1 conformational change is likely irreversible, presumably to prevent dissociated M1 from re-associating with vRNP in the cytosol after virus uncoating.

At neutral pH where basic and acidic amino acid residues exist predominantly in their ionized forms, M1 monomers are driven to interact in a face-to-back manner that involves predominantly electrostatic contacts,^10^ for example, wt-M1 1EA3 (pH 7.0) and M(NLS-88R)-neutral. At acidic pH, M1 dimerization is anticipated as it is mainly driven by hydrophobic interactions between the symmetry-related helices H6,^10^ for example, wt-M1 1AA7 (pH 4.0), M(NLS-88R)-acidic and M(NLS-88E)-acidic. Surprisingly, M(NLS-88E)-neutral dimerizes in a face-to-face manner at neutral pH in contrast to a face-to-back monomer–monomer arrangement as possessed by wt-M11EA3 (pH 7.0) and M(NLS-88R)-neutral. A further look at M(NLS-88E)-neutral structure reveals that the G88E mutation results in three inter-subunit salt-bridge/hydrogen-bond interactions including Lys104-NZ to Glu88-OE1 (and its symmetry-related counterpart) and Arg134-NE to Glu88-OE2 (Figure 1F; see Supplementary Table S3) that may explain why M(NLS-88E)-neutral that is predicted to crystallize as monomers in a face-to-back arrangement is actually driven to dimerize in a face-to-face manner at neutral pH. When compared to the M(NLS-88E)-neutral dimer interface, the positively charged Lys104 side-chain in M(NLS-88R)-acidic has reoriented to avoid close contact with the positively charged opposing Arg88 (Figures 1F and 2).

When comparing wt-M1 neutral (1EA3) and acidic (1AA7) structures, several hydrogen-bond interactions occurring at the dimer interface of 1AA7 appear to serve as the fulcrum of the subunit rotation for transition from the face-to-back-oriented 1EA3 to the face-to-face-oriented 1AA7.^10^ Some of these inter-subunit hydrogen-bond interactions are conserved in the dimeric structure of M(NLS-88)-acidic, including X88-O to Tyr100-OH and Asn85-ND2 to Arg134-O (see Supplementary Table S3). In addition to these conserved inter-subunit hydrogen-bonds, the G88R mutation also results in an extra hydrogen-bond from Arg88-NE to Arg134-O in M(NLS-88)-acidic structure. This additional inter-subunit interaction may explain why it took longer for M(NLS-88R) M1 to disintegrate *in vitro* at pH 5.5 than wt-M1, as the fulcrum of M(NLS-88R)-acidic was relatively difficult to break during the dimer interface rotation. The G88R mutation also results in two additional inter-subunit hydrogen-bonds (Lys104-NZ to Glu29-OE2 and Arg134-NE to Glu29-OE1) in M(NLS-88R)-neutral (see Supplementary Table S3) that make the subunit rotation slightly difficult than similarly face-to-back-oriented wt-M1 neutral structure 1EA3. This may explain why a lower pH was needed to soften the M1 layer in assembled M(NLS-88R) virions than in WSN particles. Nevertheless, M(NLS-88R) M1 retains pH-dependent conformational transition like WSN.

In sharp contrast to M(NLS-88R), both M(NLS-88E) neutral and acidic structures are in a face-to-face-oriented dimeric mode and lack pH-dependent conformational switches. This is because the G88E mutation causes Tyr100-OH to interact with the side chain instead of the carbonyl oxygen of mutated Glu88-OE1 (OE2), and Asn85-ND2 to interact with Arg134-NE2 instead of Arg134-O in the dimeric structure of M(NLS-88E)-neutral (see Supplementary Table S3). These changes result in a strong bifurcated hydrogen-bond interaction between Tyr100 and Glu88 that further stabilizes the three inter-subunit salt-bridge/hydrogen-bond interactions (Lys104-NZ to Glu88-OE1 and its symmetry-related counterpart, and Arg134-NE to Glu88-OE2) unique to M(NLS-88E)-neutral. This presumably makes the fulcrum in the misshapen face-to-face-oriented M(NLS-88E)-neutral very difficult to break when the pH drops, and this may explain why it requires much lower pH and takes longer time to eventually solubilize the M1 layer in assembled M(NLS-88E) virion than in M(NLS-88R) particles. As for M(NLS-88E)-acidic structure, it contains two face-to-face-arranged dimers. Dimer 2 of M(NLS-88E)-acidic is significantly different from all other obtained dimeric structures by showing neither apparent contact between Asn85 and Arg134 nor apparent inter-subunit interaction involving Glu88 (see Supplementary Table S3). Dimer 2 of M(NLS-88E)-acidic likely represents an intermediate conformation due to incomplete transition after the pH drops. However, dimer 1 of M(NLS-88E)-acidic is more like M(NLS-88E)-neutral that owns a strong bifurcated hydrogen-bond interaction between Tyr100 and Glu88 because of the G88E mutation (see Supplementary Table S3) that makes it more difficult to dissemble M(NLS-88E) M1 in acidic conditions than M(NLS-88R) M1.

Compared with M(NLS-88E), that M(NLS-88R) has less difficulty to break the fulcrum likely translates into a smaller cooperative energy requirement of monomer-to-dimer conversion under subtle pH changes.^10^ This flexibility facilitates M(NLS-88R) M1 to dissociate from vRNP in the acidified endosome, thus prompting virus replication *in vitro*. In contrast, M(NLS-88E) M1 is locked in a face-to-face-arranged dimeric conformation regardless of environmental pH. This conformational inflexibility requires much lower pH to dissociate M(NLS-88E) M1 from vRNP in the acidified endosome that not only puts M(NLS-88E) at a disadvantage in replication competency but also hinders M(NLS-88E) M1 oligomerization and genetic stability.

It is important to note that the C-terminal domain of M1, which is absent in the published crystal structures including our current study,^10, 11, 12, 13, 14^ has also been shown experimentally to influence oligomerization.^7, 8, 9^ Very recently, the crystal structure of a full-length M1 protein from a closely related Orthomyxovirus—the infectious salmon anemia virus—has just been released, showing that its C terminus plays a critical role in stabilizing M1 oligomerization.^41^ In the case of M(NLS-88E)-neutral with misshapen face-to-face monomer–monomer interactions that seem unfavorable for M1 oligomerization, a thin, uneven and broken M1 layer still forms beneath the M(NLS-88E) viral envelope as exhibited in our TEM images. This could be due to the presence of the C terminus that may provide sufficient stabilization for a malformed M1 layer. Nevertheless, the exact structural details of the full-length IAV M1 with the C terminus under different pH conditions remain to be elucidated.

Our results also indicate that the compensatory G88R mutation was not a random mutation but was chosen by the virus purposefully to retain pH-dependent conformational flexibility for efficient virus replication. Thus, critical atomic-level interactions involving position 88 of M1 are key to maintaining a delicate balance between stability and rigidity. Hence, manipulating M1 flexibility without destroying its multifunctionality can be a strategy to attenuate IAV without compromising virus replication for vaccine development.^30, 31^ Impairing M1 oligomerization through the use of a small molecule ‘wedge’ has also proven to be a viable approach to develop broad-spectrum anti-IAV agents.^42^ Besides IAV, many enveloped viruses such as *Flaviviridae* (Dengue and Zika viruses) and *Filoviridae* (Ebola and Marburg viruses) also have similar M1 structure in their viral particles. Thus, our current study offers new insights into developing M1-based countermeasures beyond IAV.

## Figures and Tables

**Table 1:** Data collection and refinement statistics of matrix protein 1 (M1) mutant structures

**Data collection statistics**	**M(NLS-88R)-acidic**	**M(NLS-88R)-neutral**	**M(NLS-88E)-acidic**	**M(NLS-88E)-neutral**
Final crystallization drop pH	pH 5.5	pH 7.3	pH 6.2	pH 7.0
Space group	P2_1_	P1	P2_1_2_1_2	P2_1_
Cell dimensions (Å)	a=39.75, b=119.82, c=59.68; β=90.2°	a=27.7, b=33.3, c=36.2; α=112.2°, β=100.4°, γ=94.2°	a=85.61, b=133.26, c=39.31	a=40.19, b=96.17, c=48.49; β=100.9°
Resolution (Å)	29.95–2.0 (2.07–2.0)	28.70–3.0 (3.11–3.00)	26.23–2.5 (2.59–2.5)	27.91–2.5 (2.59–2.5)
Measured reflections	205147	6194	79188	44337
Unique reflections	33892 (3432)	2187 (247)	14742 (1473)	12404 (1227)
Redundancy	6.05 (5.97)	2.83 (2.97)	5.37 (5.51)	3.57 (3.5)
I/σI	13.1 (5.9)	10.9 (1.5)	12.1 (3.6)	9.5 (2.7)
Completeness (%)	90.0 (91.9)	94.5 (93.2)	90.7 (91.9)	98.7 (99.3)
*R*_merge_ (%)^a^	8.8 (27.8)	8.1 (51.2)	7.7 (44.9)	7.2 (37.9)
				
*Structure refinement*				
Resolution limit (Å)	29.84–2.00 (2.07–2.0)	21.93–3.00 (3.23–3.00)	26.23–2.50 (2.69–2.50)	27.58–2.50 (2.59–2.50)
No. of reflections	33843 (3428)	2177 (427)	14714 (1470)	12376 (1172)
*R*_work_ (%)	19.2 (24.0)	27.2 (39.7)	22.0 (30.2)	22.8 (34.3)
*R*_free_ (%)^b^	23.6 (30.5)	32.1 (43.4)	31.2 (41.7)	30.8 (31.0)
				
*R.m.s.d. geometry*				
Bond lengths (Å)	0.006	0.003	0.009	0.008
Bond angles	1.1°	0.595°	1.165°	1.3°
				
*Dihedral angles (%)*				
Most favored	96.46	93.66	92.22	92.83
Allowed regions	2.09	5.63	5.83	2.93
				
*Av. B-factors (Å*^*2*^)				
All atoms	26.10	80.20	56.90	61.00
Protein alone	24.10	80.20	56.80	61.00
Water	36.80	69.20	47.50	58.00
PDB ID	5V6G	5V8A	5V7S	5V7B

a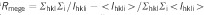.

b *R*_free_ was calculated with 5% excluded reflection from the refinement.
