# Supplementary material for: Maintaining pH-dependent conformational flexibility of M1 is critical for efficient influenza A virus replication
Source: Emerg Microbes Infect. 2017 Dec 6;6(12):e108–. doi: 10.1038/emi.2017.96 (PMC5750462; doi:10.1038/emi.2017.96)

Supplementary Figure S1 Effects of different pHs on cellular membrane associated M1. (A) Representative Western Blots imaged using an Odyssey imaging system. (B) Ratio of membrane-associated M1 to β-actin following different pH treatments (n= 3). The intensity of each protein band probed by specific antibody was measured using an Odyssey imaging system.

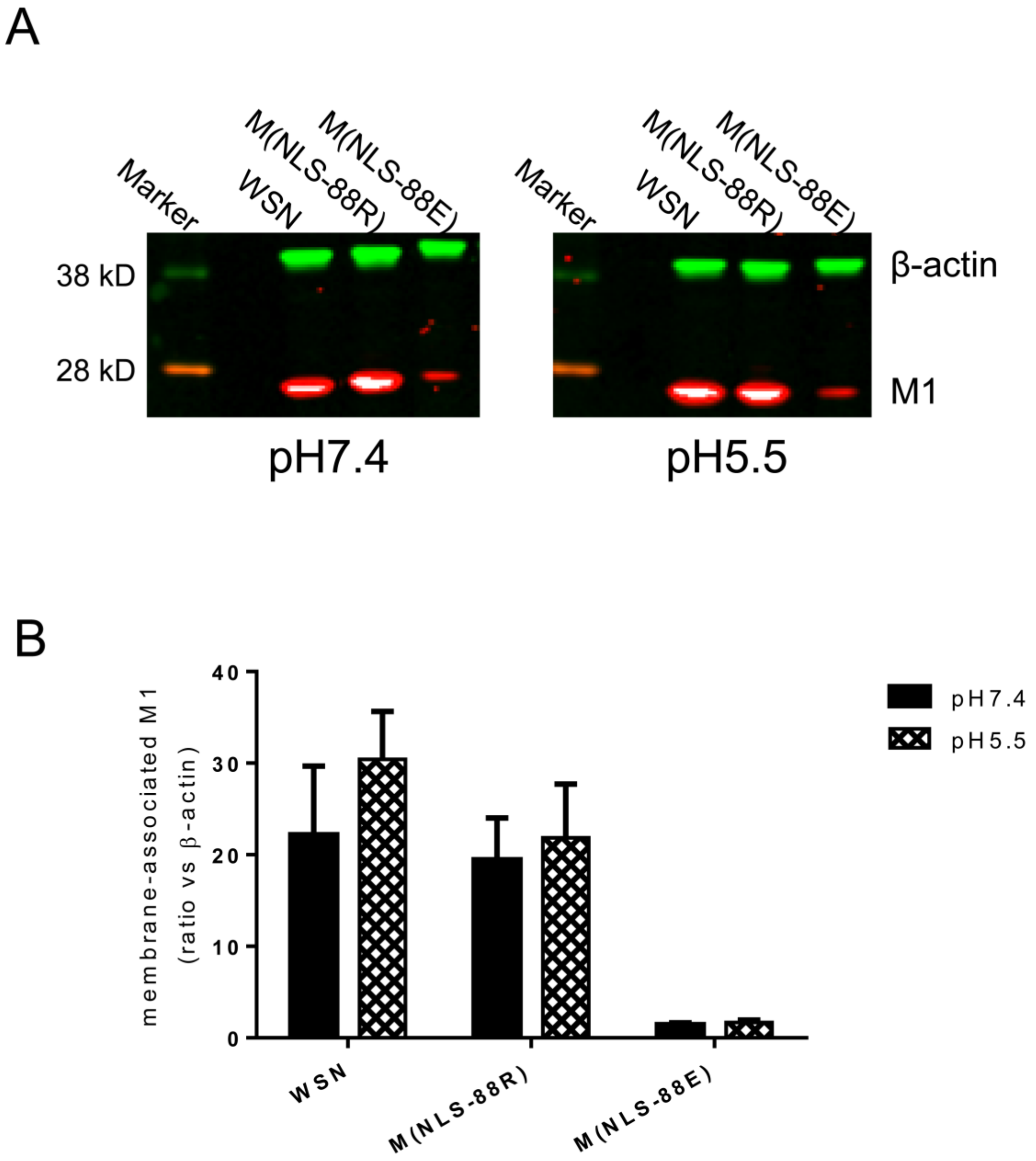

Supplement: Supplementary Figure S1 [file emi201796x1.pdf]
