# Supplementary material for: Maintaining pH-dependent conformational flexibility of M1 is critical for efficient influenza A virus replication
Source: Emerg Microbes Infect. 2017 Dec 6;6(12):e108–. doi: 10.1038/emi.2017.96 (PMC5750462; doi:10.1038/emi.2017.96)

Supplementary Figure S2 Enhanced image view of pH-dependent M1–NP co-localization using Imaris Image Analysis Software. Wild type A/WSN/33 (WSN), M(NLS-88R) or M(NLS-88E) pre-treated with different pH conditions were bound to pre-chilled MDCK cells on ice for 60 min followed by acid bypass to allow direct membrane fusion. Immunofluorescent stained M1 (green) and NP (red) captured by FluoView® FV10i Confocal Laser Scanning Microscope shown in the main Figure 5 were reprocessed using Imaris Image Analysis Software (Bitplane) for spot counting (A–L). Nuclei (yellow) are excluded from spot identification because of high background. (M) Total M1 & NP spots counted in reprocessed image A-L.

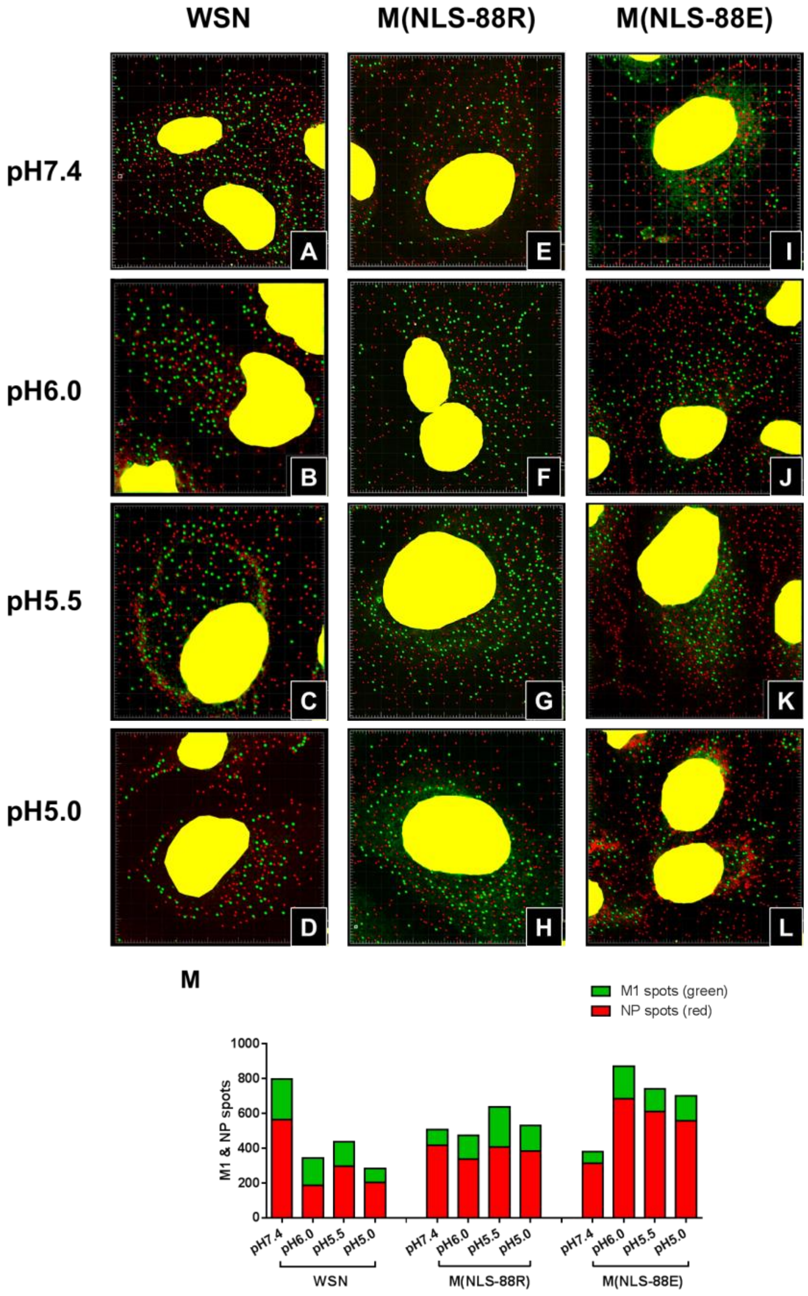

Supplement: Supplementary Figure S2 [file emi201796x2.pdf]
