# Supplementary material for: Maintaining pH-dependent conformational flexibility of M1 is critical for efficient influenza A virus replication
Source: Emerg Microbes Infect. 2017 Dec 6;6(12):e108–. doi: 10.1038/emi.2017.96 (PMC5750462; doi:10.1038/emi.2017.96)
Supplement: Supplementary Table S1 [file emi201796x3.doc]

**Supplementary Table S1** Genetic stability of M1 mutants after being serially passaged in embryonic eggs^a^

| **Virus** | **Egg Passage** | **Amino Acid Position of M1** | | | | | | | | |
| --- | --- | --- | --- | --- | --- | --- | --- | --- | --- | --- |
|  |  | **75** | **88** | **94** | **101** | **105** | **110** | **144** | **147** | **153** |
| WSN | E1-E10 | Q | G | D | R | R | H | F | V | Q |
| M(NLS-88R) | E1-E10 | - | *R* | *-* | *S* | *S* | - | - | - | - |
|  | E11 | - | *R* | *-* | *S* | *S* | - | *-* | *-* | *Q/H* |
|  | E12 | - | *R* | *-* | *S* | *S* | - | *-* | *-* | *H* |
| M(NLS-88E) | E1-E3 | - | *E* | *-* | *S* | *S* | - | - | - | - |
|  | E4-E11 | *K* | *E* | *-* | *S* | *S* | *Y* | - | - | - |

**^a^**A/WSN/33 (WSN) and its M1 mutants were generated by reverse genetics. Both M(NLS-88R) and M(NLS-88E) bear the R101S and R105S mutations in the nuclear localization signal motif (NLS) of M1 and differ in a third mutation at M1 position 88 (G88R or G88E). “-” indicates no change.
