# Supplementary material for: Maintaining pH-dependent conformational flexibility of M1 is critical for efficient influenza A virus replication
Source: Emerg Microbes Infect. 2017 Dec 6;6(12):e108–. doi: 10.1038/emi.2017.96 (PMC5750462; doi:10.1038/emi.2017.96)
Supplement: Supplementary Table S2 [file emi201796x4.doc]

**Supplementary Table S2 Tertiary and quaternary structure differences of the M1 dimer structures^a^**

| **Compared structures** | **Monomer (Å)^b^** | **Dimer (Å)^c^** | **Screw-rotation angle (°) /translation (Å)^d^** |
| --- | --- | --- | --- |
| 1AA7 (wt, acidic) ↔ M(NLS-88R)-acidic | 0.6 | 0.6 | 2/0.1 |
| 1AA7 (wt, acidic) ↔ M(NLS-88E)-acidic (Dimer1) | 0.6 | 1.4 | 10/0.4 |
| 1AA7 (wt, acidic) ↔ M(NLS-88E)-acidic (Dimer2) | 0.5 | 0.7 | 4/0.0 |
| 1AA7 (wt, acidic) ↔ M(NLS-88E)-neutral | 0.7 | 1.9 | 18/0.2 |
| M(NLS-88R)-acidic ↔ M(NLS-88E)-acidic (Dimer2) | 0.4 | 0.5 | 2/0.2 |
| M(NLS-88R)-acidic ↔ M(NLS-88E)-neutral | 0.6 | 1.8 | 16/0.3 |
| M(NLS-88E)-acidic (Dimer1) ↔ M(NLS-88E)-acidic (Dimer2) | 0.6 | 1.2 | 8/0.2 |
| M(NLS-88E)-neutral ↔ M(NLS-88E)-acidic (Dimer1) | 0.6 | 1.1 | 8/0.1 |
| M(NLS-88E)-neutral ↔ M(NLS-88E)-acidic (Dimer2) | 0.6 | 1.8 | 15/0.2 |

^a^See text for a detailed structural comparison. ^b^Least-squares superpositions of monomers. ^c^Least-squares superpositions of dimers. ^d^Superposition of monomers A, and the rigid-body screw rotation that aligns the non-superposed monomers B.
