# Supplementary material for: Maintaining pH-dependent conformational flexibility of M1 is critical for efficient influenza A virus replication
Source: Emerg Microbes Infect. 2017 Dec 6;6(12):e108–. doi: 10.1038/emi.2017.96 (PMC5750462; doi:10.1038/emi.2017.96)
Supplement: Supplementary Table S3 [file emi201796x5.doc]

**Supplementary Table S3** Hydrogen-bond interactions (<3.8 Å) and solvent-accessible buried surface area of the M1 monomer A–monomer B interface. Interacting amino acids shown in red indicate those positions common to all face-to-face interfaces (see main text for details)

|  | **Monomer A** | **Monomer B** | **Distance (Å)** | **Buried Surface Area (Å^2^)** |
| --- | --- | --- | --- | --- |
| **Dimer Structures** | | | | |
| **wt-M1**  **(1AA7, acidic)** | Gln75(OE1) | Arg78(N) | 2.9 | 2187 |
|  | Arg78(N) | Gln75(OE1) | 3.0 |  |
|  | Arg76(O) | Gln75(NE2) | 3.2 |  |
|  | Gln81(OE1) | Arg134(NE) | 3.1 |  |
|  | Gly88(O) | Tyr100(OH) | 2.9 |  |
|  | Tyr100(OH) | Gly88(O) | 2.7 |  |
|  | Arg101(NH2) | Asn91(OD1) | 3.0 |  |
|  | Asn91(OD1) | Arg101(NH1) | 3.4 |  |
|  | Asn133(O) | Asn85(ND2) | 3.1 |  |
|  | Asn85(ND2) | Arg134(O) | 3.1 |  |
|  | Arg134(O) | Asn85(ND2) | 3.2 |  |
|  | Gln75(NE2) | Arg77(NH1) | 3.4 |  |
|  | Asn133(ND2) | Asn133(O) | 3.6 |  |
|  | | | | |
| **M(NLS-88R)-acidic** | Gly73(O) | Arg78(NH1) | 3.6 | 2327 |
|  | Gln75(OE1) | Arg76(O) | 3.2 |  |
|  | Gln75(OE1) | Arg78(N) | 2.7 |  |
|  | Gln75(NE2) | Arg77(NH1) | 3.6 |  |
|  | Arg76(O) | Gln75(OE1) | 3.2 |  |
|  | Arg77(NH1) | Gln75(NE2) | 3.4 |  |
|  | Arg78(N) | Gln75(OE1) | 3.0 |  |
|  | Gln81(OE1) | Arg134(NE) | 2.7 |  |
|  | Arg134(NH1) | Gln81(OE1) | 3.1 |  |
|  | Gln81(NE2) | Gln81(NE2) | 3.3 |  |
|  | Asn85(ND2) | Arg134(O) | 3.4 |  |
|  | Arg88(NH1) | Arg134(O) | 3.1 |  |
|  | Arg134(O) | Arg88(NE) | 3.4 |  |
|  | Arg88(O) | Tyr100(OH) | 3.1 |  |
|  | Tyr100(OH) | Arg88(O) | 3.5 |  |
|  | Met93(SD) | Met135(O) | 3.6 |  |
|  | Asn133(O) | Asn133(ND2) | 3.6 |  |
|  | | | | |
| **M(NLS-88E)-acidic**  **(Dimer 1)** | Asn85(ND2) | Arg134(NE) | 3.4 | 1279 |
|  | Asn85(ND2) | Arg134(NH2) | 3.1 |  |
|  | Glu88(O) | Tyr100(OH) | 3.2 |  |
|  | Tyr100(OH) | Glu88(OE1) | 2.7 |  |
|  | Tyr100(OH) | Glu88(OE2) | 3.4 |  |
|  | Lys104(NZ) | Glu88(OE1) | 3.4 |  |
|  | Lys104(NZ) | Glu88(O) | 3.4 |  |
|  | Arg134(NH1) | Glu88(OE2) | 2.2 |  |
|  | Asn134(NH2) | Asn85(OD1 | 3.0 |  |
|  | Asn133(O) | Gln81(NE2) | 3.2 |  |
|  | Asn133(O) | Gln81(OE1) | 3.2 |  |
|  | Met93(SD) | Arg134(NH1) | 3.6 |  |
|  | Asp94(OD1) | Lys98(NZ) | 3.3 |  |
|  | | | | |
| **M(NLS-88E)-acidic**  **(Dimer 2)** | Gly73(O) | Arg78(NH2) | 3.4 | 1382 |
|  | Ala74(O) | Arg78(NH2) | 2.9 |  |
|  | Arg78(NH2) | Gly73(O) | 3.4 |  |
|  | Arg78(NH2) | Ala74(O) | 2.9 |  |
|  | Gln75(OE1) | Arg76(O) | 3.3 |  |
|  | Gln75(OE1) | Arg78(N) | 2.6 |  |
|  | Gln75(NE2) | Arg77(NE) | 3.3 |  |
|  | Arg76(O) | Gln75(OE1) | 3.3 |  |
|  | Arg77(NE) | Gln75(NE2) | 3.3 |  |
|  | Arg78(N) | Gln75(OE1) | 2.6 |  |
|  | Arg78(NE) | Arg134(NH2) | 2.9 |  |
|  | Arg134(NH2) | Arg78(NE) | 2.9 |  |
|  | Gln81(OE1) | Arg134(NE) | 2.6 |  |
|  | Arg134(NE) | Gln81(OE1) | 2.6 |  |
|  | Glu88(O) | Tyr100(OH) | 2.7 |  |
|  | Tyr100(OH) | Glu88(O) | 2.7 |  |
|  | Asn133(ND2) | Asn133(O) | 2.7 |  |
|  | Asn133(O) | Asn133(ND2) | 2.7 |  |
|  | | | | |
| **M(NLS-88E)-neutral** | Asn85(ND2) | Arg134(NE) | 3.3 | 1264 |
|  | Glu88(OE1) | Tyr100(OH) | 3.0 |  |
|  | Glu88(OE2) | Tyr100(OH) | 3.0 |  |
|  | Glu88(OE2) | Lys104(NZ) | 2.8 |  |
|  | Lys104(NZ) | Glu88(OE1) | 2.7 |  |
|  | Tyr100(OH) | Glu88(OE1) | 2.8 |  |
|  | Tyr100(OH) | Glu88(OE2) | 2.7 |  |
|  | Arg134(NE) | Glu88(OE2) | 3.5 |  |
| **Monomer Structures** | | | | |
| **wt-M1**  **(1EA3, neutral)** | Asp89 (OD1) | Asn36(ND2) | 3.5 | 896 |
|  | Asp94(OD2) | Lys21(NZ) | 3.2 |  |
|  | Asp94(OD2) | Ser17(OG) | 3.1 |  |
|  | Tyr100(OH) | Glu29(OE1) | 3.5 |  |
|  | Arg101(NH2) | Glu8(OE1) | 3.4 |  |
|  | Arg101(NH2) | Glu8(OE2) | 3.4 |  |
|  | Lys104(NZ) | Glu29(OE1) | 3.1 |  |
|  | Arg134(NH1) | Asp30(OD1) | 2.7 |  |
|  | Arg134(NH1) | Asp30(OD2) | 3.4 |  |
|  | Arg134(NH2) | Asp30(OD1) | 3.1 |  |
|  | Arg134(NH2) | Asp30(OD2) | 2.5 |  |
|  | | | | |
| **M(NLS-88R)-neutral** | Asp94(OD1) | Lys21(NZ) | 2.5 | 851 |
|  | Asp94(OD1) | Ser17(OG) | 2.6 |  |
|  | Tyr100(OH) | Glu29(OE1) | 2.9 |  |
|  | Lys104(NZ) | Glu29(OE1) | 3.4 |  |
|  | Lys104(NZ) | Glu29(OE2) | 2.8 |  |
|  | Arg134(NE) | Glu29(OE1) | 3.5 |  |
|  | Arg134(NH1) | Glu29(OE1) | 2.6 |  |
|  | Arg134(NH2) | Asp30(OD1) | 2.2 |  |
|  | Arg134(NH2) | Asp30(OD2) | 3.4 |  |
